# The Role of Inflammation and Immune Cells in Blood Flow Restriction Training Adaptation: A Review

**DOI:** 10.3389/fphys.2018.01376

**Published:** 2018-10-09

**Authors:** Fabrício Eduardo Rossi, Marcelo Conrado de Freitas, Nelo Eidy Zanchi, Fábio Santos Lira, Jason M. Cholewa

**Affiliations:** ^1^Exercise and Immunometabolism Research Group, Department of Physical Education, São Paulo State University (UNESP), Presidente Prudente, Brazil; ^2^Skeletal Muscle Assessment Laboratory, Department of Physical Education, School of Technology and Sciences, São Paulo State University, Presidente Prudente, Brazil; ^3^Laboratory of Cellular and Molecular Biology of Skeletal Muscle (LABCEMME), Department of Physical Education, Federal University of Maranhão (UFMA), São Luís, Brazil; ^4^Department of Kinesiology, Coastal Carolina University, Conway, SC, United States

**Keywords:** immune cell, occlusion, hypertrophy, resistance exercise, KAATSU training

## Abstract

Blood flow restriction (BFR) combined with low-intensity strength training has been shown to increase skeletal muscle mass and strength in a variety of populations. BFR results in a robust metabolic stress which is hypothesized to induce muscle growth via increased recruitment of fast-twitch muscle fibers, a greater endocrine response, and/or enhancing the cellular swelling contribution to the hypertrophic process. Following exercise, neutrophils are the first immune cells to initiate the tissue remodeling process via several mechanisms including an increased production of cytokines and recruitment of monocytes/macrophages, which facilitate the phagocytosis of foreign particles, the differentiation of myoblasts, and the formation of new myotubes. Thus, the purpose of this review was to discuss the mechanisms through which metabolic stress and immune cell recruitment may induce skeletal muscle remodeling following BFR strength training.

## Introduction

Strength training has been the classically employed exercise program to increase skeletal muscle hypertrophy and strength. The *American College of Sports Medicine* (Evetovich, 2009) recommends strength training with intensities over 70% of the one repetition maximum (1RM) to increase muscle mass, and several studies have shown that intensities in excess of 70% 1RM greater stimulate muscle protein synthesis, satellite cell activity, and decrease proteolysis when compared to low-intensity resistance exercise (Harridge, 2007; Louis et al., 2007). There is a growing body of evidence, however, that lower-intensity strength training induces similar degrees of muscle protein synthesis acutely (Burd et al., 2010, 2012) and muscle hypertrophy chronically (Schoenfeld et al., 2016) when sets are carried out to muscular failure. Although heavier loading seems necessary to maximize muscular strength development (Peterson et al., 2005), high-intensity loads performed in traditional strength training programs may not be a viable option for sarcopenic populations, older individuals with chronic health problems, or individuals with injuries who may be unable to tolerate heavy loading.

Blood flow restriction (BFR), also known as occlusion or KAATSU training (Abe et al., 2005), combined with low-intensity strength training (20–30% 1RM) has been shown to increase muscle size and strength in the elderly (Libardi et al., 2015), athletes (Takarada et al., 2002), untrained (Clark et al., 2011), and people with injuries (Loenneke et al., 2013). This training model requires the use of inflatable cuffs that are placed at the proximal ends of the upper arms or thighs to restrict blood flow, whereby the external pressure applied is sufficient to maintain arterial inflow whilst occluding venous outflow distal to the occlusion site (Kaijser et al., 1990), thus resulting in an ischemic/hypoxic environment that enhances the training effect in exercising muscle (Takada et al., 2012).

Several studies have compared low-intensity strength training with BFR and high-intensity strength training without BFR and demonstrated similar increases in muscle cross-section area between exercise protocols (Ellefsen et al., 2015; Farup et al., 2015; Libardi et al., 2015), however, the mechanisms responsible for such effects are still poorly understood. Mechanical tension and metabolic stress are suggested to be the factors primarily responsible for hypertrophic adaptations following BFR training (Pearson and Hussain, 2015).

The mechanisms by which mechanical tension induces muscle hypertrophy include mechanotransduction, where sarcolemmal-bound mechanosensors (i.e., integrins) stimulate intracellular anabolic and catabolic pathways which convert mechanical energy into chemical signals promoting protein synthesis instead of degradation (Zou et al., 2011). Exercise-induced muscle damage can be influenced by an increase in the volume, velocity, initial muscle length, or the intensity of the exercise. These stimuli can result in an overstretching of the sarcomere to such an extent that it becomes disrupted, resulting in z-disk damage and eventually a disruption of the cytoskeletal matrix (Proske and Morgan, 2001). This may promote activation of the stretch-activated calcium channels or transient receptor potential channels (Allen et al., 2005). Consequently, cytokines are released and there is an immune system activation, directing the local satellite cells to the site of muscle damage and initiating the phagocytosis of debris and tissue remodeling (Tidball and Villalta, 2010).

In contrast to the muscle damage that occurs as a result of traditional strength training, a review conducted by Loenneke et al. (2014a) does not support the hypothesis that BFR training increases the incidence of muscle damage. Sudo et al. (2015) verified muscle fiber damage following high-intensity eccentric contractions and BFR plus eccentric contractions in male Wistar rats with different levels of occlusion pressure (140, 160, and 200 Torr). Despite BFR increased Ribosomal S6 kinase 1 (S6K1) phosphorylation, a downstream target of rapamycin (mTOR)-phosphorylation kinase, there was no measurable muscle damage for any of the three different pressures (2.6 ± 1.2%), (3.0 ± 0.5%), (0.2 ± 0.1%), respectively, compared to high-intensity (26.4 ± 4.0%) when verified the histochemical muscle fiber damage (area of damaged fibers/total fiber area analyzed). In humans, Neto et al. (2018) compared low-load resistance exercise (20% 1RM) plus continuous or intermittent BFR and high-intensity resistance exercise (80% 1RM) and observed higher creatine kinase and lactate dehydrogenase after high-intensity compared with both continuous or intermittent exercise with BFR 24 and 48 h post-exercise. In addition, Wilson et al. (2013) demonstrated significantly increases muscle activation and muscle thickness, but no changes in muscle soreness, power, and muscle swelling after 4 sets of leg press (30-15-15-15-repetition at 30% 1 RM) in trained men. Collectively, this research refutes the muscle damage hypothesis post-BFR training.

**Figure 1:**
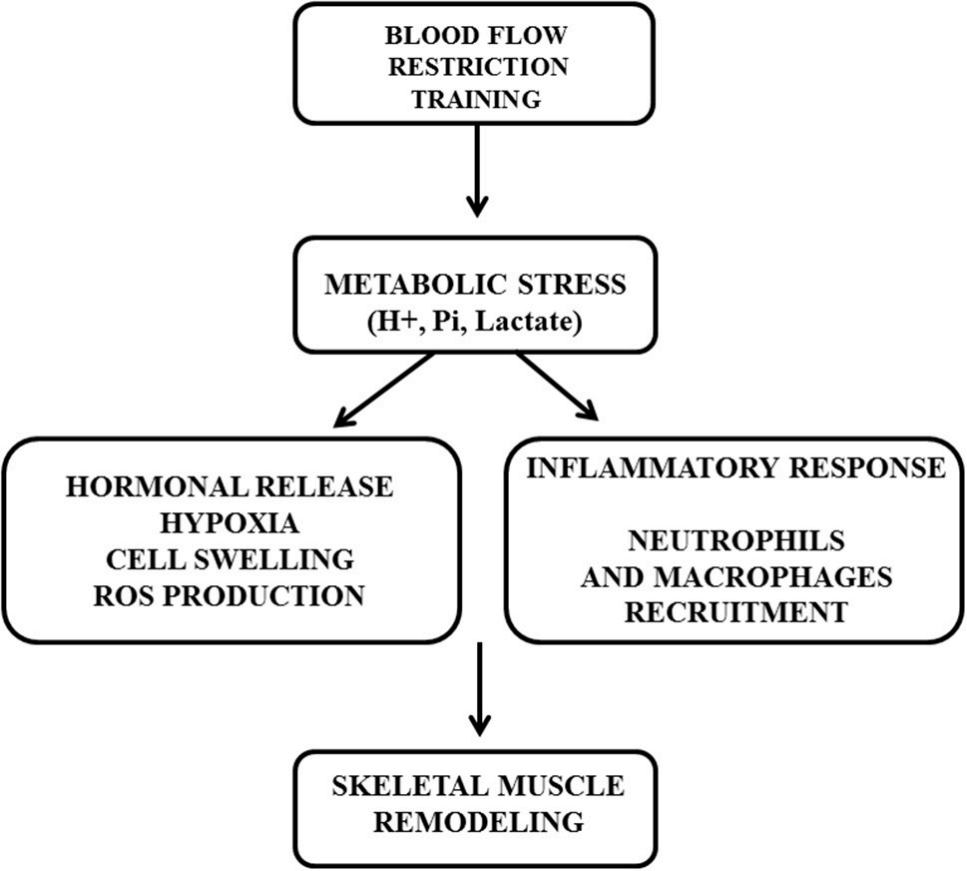
The role of metabolic stress (H+, Pi, Lactate) leading to anabolic signaling (hormonal release, hypoxia, cell swelling, ROS production) and inflammatory response induced by blood flow restriction training for enhancing muscle adaptations.

In this sense, the metabolic stress during BFR as a result of the ischemic/hypoxic environment seems to be the prevailing mechanism whereyby BFR could potentiate hypertrophy during low-intensity strength training. Metabolic stress and/or hypoxia may leads to an increased recruitment of fast-twitch muscle fibers (Suga et al., 2009), increased inflammatory and endocrine response (Fujita et al., 2007), cellular swelling (Loenneke et al., 2013), and elevated intramuscular inorganic phosphates (Takada et al., 2012), all of which have been shown mediate muscle protein signaling and satellite cell proliferation (Figure 1).

Hypoxia-inducible factor-1 alpha (HIF-1α) activation contributes to an increased expression of angiogenic factors, vascular endothelial growth factor (VEGF), endothelial nitric oxide synthase (eNOS) (Larkin et al., 2012), and monocyte chemotactic protein-1 (MCP-1). Warren et al. (2004) used a freeze injury model to evaluate muscle repair and inflammation in mice and observed a significant importance for MCP-1, macrophage inflammatory protein (MIP)-1alpha, MIP-1beta, and monocyte chemoattractant protein during muscle repair. Mice that were null mutants for MCP-1 were found to have a slowed recovery from muscle injury compared to wild-type animals as assessed by muscle force production. Shill et al. (2017) observed that BFR using unilateral isometric wrist exercise increased concentrations of fibroblast growth factor, IL-6, IL-10, TNFα, and VEGF during exercise and reduced the percentage of oxygen saturation, demonstrating that the hypoxic environment generated during BFR was associated with a heightened inflammatory cytokine production.

Thus, as hypothesized by Pearson and Hussain (2015), both mechanical tension and metabolic stress would synergistically contribute to the hypertrophic adaptations of BFR strength training, with metabolic stress playing the dominant role. Although the efficacy of BFR strength training to induce skeletal muscle hypertrophy has been well demonstrated in the literature (Loenneke and Pujol, 2009; Patterson and Ferguson, 2011; Takada et al., 2012; Loenneke et al., 2013; Pope et al., 2013), the mechanism by which BFR results in an inflammatory response and immune cell recruitment is still poorly understood. Therefore, the purpose of this review is to discuss the relationship between metabolic stress and immune cell recruitment in skeletal muscle remodeling following BFR strength training.

## The inflammatory response during blood flow restriction training: potential mechanisms

The stress induced by exercise results in an inflammatory response mediated by an increase in myokine production, which are one of several hundred cytokines and proteoglycan peptides that are produced and released by muscle cells (myocytes) in response to muscular contractions (Giudice and Taylor, 2017). Myokines, specifically IL-6, play a role in skeletal muscle tissue regeneration after damaging exercise, in part by participating in the recruitment of neutrophil, monocytes, and lymphocytes, which phagocytize debris materials (Vannella and Wynn, 2017). In addition, IL-6 also contributes to the activation, differentiation and proliferation of satellite cells, which migrate and fuse to the spaces where muscle fiber damage occurred and contribute new myonuclei (Serrano et al., 2008). Recently, Gao et al. (2017) investigated the influence of IL-6 on mTORC1 signaling and protein synthesis in cultured myotubes. It was observed that IL-6 can increase protein synthesis by activating the gp130-Akt and mTORC1 pathway in a dose-sensitive manner, suggesting that the IL-6 response to resistance exercise potentiates muscle hypertrophy.

Regarding the influence of BFR on the inflammatory response, Patterson and Ferguson (2011) compared the effects of BFR (five sets of unilateral knee extensions with 20% 1-RM and 30-s rest between each set) with low-load resistance exercise without BFR older men. The authors observed that both conditions increased serum IL-6 at 60 and 120 min post-exercise, but GH and VEGF only increased following the BFR condition. Takarada et al. (2000) also compared the IL-6 response after BFR using five sets of 14 repetitions at 20% of 1-RM vs. the same exercise protocol without occlusion (control). The results showed that plasma IL-6, lactate, GH and norepinephrine was greater in BFR than the control condition without any increase in CK activity 24 h post-exercise in either condition, suggesting that BFR may induce a higher metabolic stress (accumulation of lactate) and hormonal response without apparent markers of muscle damage. Supporting this, Nielsen et al. (2017) showed that short-term BFR (unilateral knee extensor exercise to failure at 20% 1-RM) generated modest increases in the inflammatory responses (MCP-1, IL-6, and TNF-α) with lower muscle damage.

According to these findings it appears that the early increase in IL-6 in response to BFR may be in part due to a greater metabolic stress more so than muscle damage. Studies consistently demonstrate that BFR generates a high concentration of lactate accumulation, H+, and a significant decrease in intramuscular pH, suggesting that BFR is highly reliant on rapid glycolytic metabolism (Takarada et al., 2000; Suga et al., 2009, 2012). Previous studies also reported an association between anaerobic metabolism and an increase in the production of inflammatory cytokines (Pedersen and Febbraio, 2005). Additionally, the reduction of muscle glycogen during exercise seems to potentiate the IL-6 response (Pedersen and Febbraio, 2005), and acute ischemic exercise induced by BFR results in a higher degree of glycogen depletion of type II fibers compared with non-ischaemic exercise (Sundberg, 1994). In this context, the increased IL-6 response by BFR may be explained by the predominance of anaerobic glycolytic metabolism and glycogen depletion.

**Figure 2:**
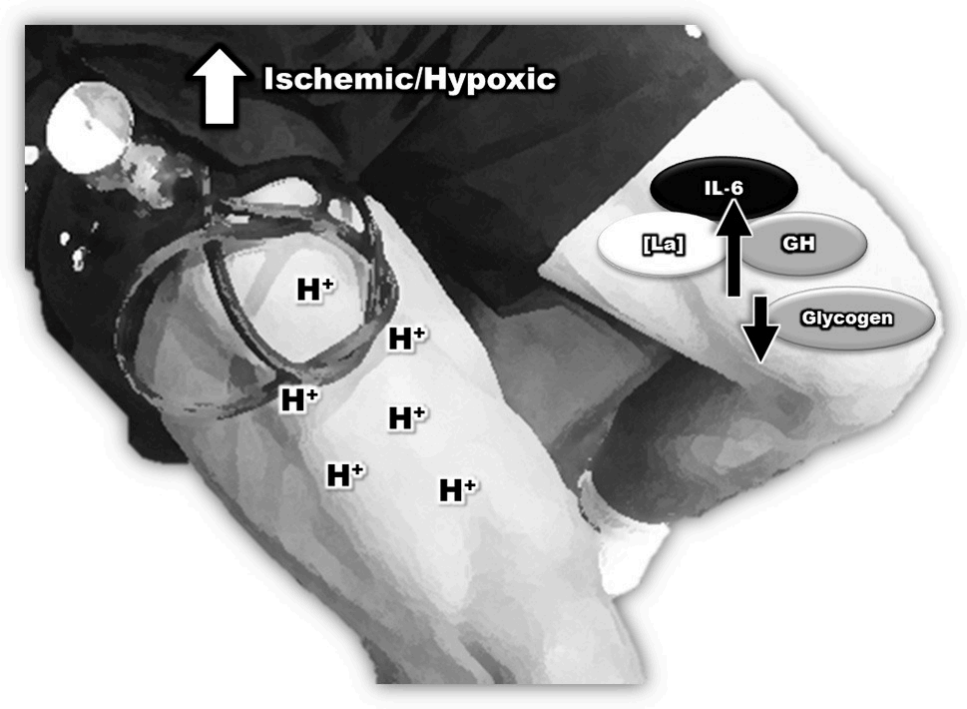
The inflammatory response during blood flow restriction plus low-intensity strength training.

Another possible driver of the IL-6 response during BFR could be attributed to intramuscular hypoxia. It has been reported that BFR decreases muscle oxygenation, generating a hypoxic environment (Karabulut et al., 2011; Ganesan et al., 2015) leading to a robust activation of HIF-1α. Several studies have demonstrated that HIF-1α has the potential to increase pro-inflammatory gene expression, such as IL-6 and TNF-α, via increasing nuclear factor kappa B (NF-kB) translocation to the cell nucleus (Van Uden et al., 2008; Oliver et al., 2009; Szade et al., 2015). An increased expression of HIF-1α has also been associated with a high production of inflammatory cytokines (IL-6 and TNF-α). Leire et al. (2013) reported that when cell cultures were treated with AKB-4924, an activator of HIF-1α, IL-6 levels significantly increased, demonstrating the influence of HIF-1α on IL-6 production. In support of this hypothesis, Drummond et al. (2008) found that bilateral knee extension exercise with BFR (4 sets of 30, 15, 15, 15 repetition at 20% of 1RM, with 30 s rest period) increased HIF-1α gene expression. Shill et al. (2017) observed that BFR using unilateral isometric wrist exercise increased concentrations of fibroblast growth factor, IL-6, IL-10, TNFα, and VEGF and reduced the percentage of oxygen saturation, demonstrating a link between the BFR induced hypoxic environment and cytokine production (Figure 2).

In summary, studies have demonstrated that BFR increases the IL-6 concentrations absent of significant muscle damage, suggesting that other mechanisms likely regulates the IL-6 response to BFR. It seems that the metabolic stress associated with glycogen depletion and hypoxia could be a potential mechanism by which BFR modulates the acute immune response.

## Neutrophil and macrophage response to resistance exercise

Multiple immune cell types play a critical role in muscle repair, regeneration and remodeling following exercise. Neutrophils, macrophages and T cells seem to be the first immune cells to initiate the recovery process following exercise-induced muscle damage. Neutrophils constitute 60% of the circulating leukocytes and are the most abundant cellular component of the human immune system, with a total number of approximately 5 × 10^11^ cells in a 70 kg individual (Pyne, 1994). It has been suggested (Pyne, 1994) that neutrophil recruitment is an essential role in the initial stages of muscle repair and regeneration after exercise, as neutrophils act to clear cellular debris via the release of proteolytic enzymes for several hours after exercise.

Neutrophils accumulation in skeletal muscle likely starts immediately after exercise, as Frenette et al. (2000) showed a 60% increase in neutrophil invasion 2 h after muscle injury. Previous studies demonstrated neutrophil accumulation in the blood vessels of skeletal muscle occurs after 1 and 24 h after eccentric exercise (Raastad et al., 2003; Paulsen et al., 2010), and histological studies have demonstrated that after 24–48 h of exercise, there is an increase of leukocytes infiltratation in the extracellular space within the muscle (Hellsten et al., 1997; Paulsen et al., 2010). Animal studies have also demonstrated a clear influence of neutrophils on muscle regeneration after exercise, as depletion of neutrophils in mice before muscle injury impairs skeletal muscle regeneration, likely as a result of a reduced capacity to remove tissue debris by phagocytes that slowed regenerative process (Teixeira et al., 2003).

After the acute neutrophil accumulation, macrophages, a subpopulation of leukocytes, infiltrate the muscle and play an important role in the later stages of muscle repair and regeneration. The monocytes/macrophages are attracted in skeletal muscle tissue by chemotactic factors such as fractalkine (CX3CL1) and monocyte chemotactic protein-1 (MCP-1), contributing to skeletal muscle repair by phagocytosis of foreign particles, secreting pro-inflammatory cytokines (e.g., TNFα and IL-6) and stimulating satellite cell proliferation (Peake and Neubauer, 2017). Previous studies report that the increase in macrophages in human skeletal muscle occurred at later time points in recovery, around at 48 h to 7 days after exercise (Jones et al., 1986; Marklund et al., 2013). It has been demonstrated that macrophages play a critical role for muscle regeneration, as inhibiting the recruitment of monocytes to injured muscles impairs muscle regeneration (Lu et al., 1998). Warren et al. (2004) used a freeze injury model to evaluate muscle repair and inflammation in mice and observed a significant importance for MCP-1 and macrophage recruitment to potentiate skeletal muscle regeneration. Mice that were null mutants for Chemokine Receptor 5 (CCR5) and MCP-1 were found to have slowed recovery from muscle injury compared to wild-type animals as assessed by muscle force production as an index of recovery.

**Figure 3:**
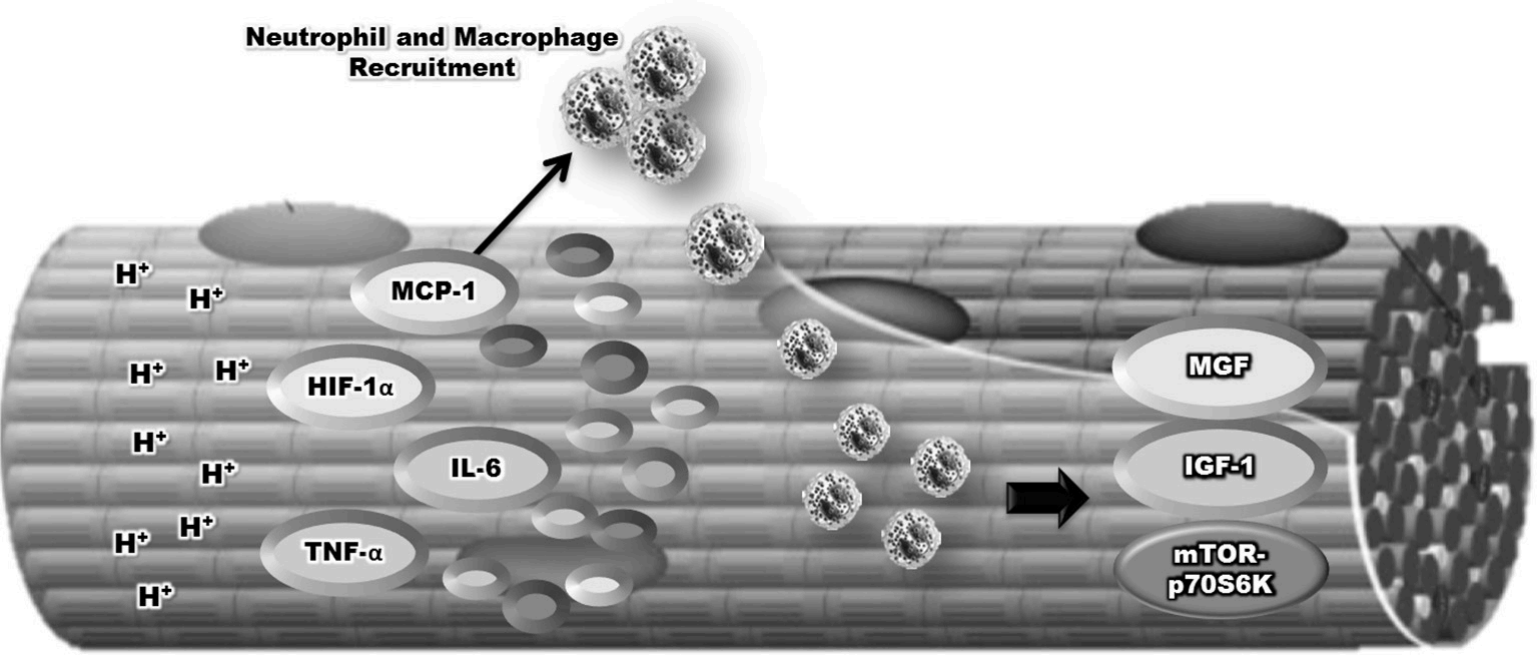
Relationship between inflammatory, neutrophil, and macrophage in the remodeling of skeletal muscle mass induced by blood flow restriction plus strength training.

Additionally, both macrophages and neutrophils appear to mediate muscle hypertrophy by the secretion of pro-inflammatory cytokines (IL-6 and TNFα), leading to anabolic signaling through a pathway that phosphorylates mTORC1 (Schoenfeld et al., 2016). It has been demonstrated that the production of ROS may stimulate anabolic signaling, as ROS can act with a signaling molecule to activate mTORC1 signaling via the IGF-1 and MAPK pathways (Kefaloyianni et al., 2006; Handayaningsih et al., 2011). In addition, neutrophils and macrophages can release growth factors such as insulin-like growth factor (IGF-1), basic Fibroblast Growth Factor (bFGF), Transforming Growth Factor (TGF), and Mechano Growth Factor (MGF), and these factors contribute to the muscle regeneration and hypertrophic response by activating satellite cells and mTORC1 signaling (Jiang et al., 1992; Cantini et al., 2002; Koh and Pizza, 2009; Zanou and Gailly, 2013; Figure 3).

## Neutrophil and macrophage response to blood flow restriction exercise

After traditional resistance exercise there is an increase in neutrophils (Macintyre et al., 2000; Simonson and Jackson, 2004) and macrophages (Malm et al., 1999); however, the influence of BFR on the immune cell response is less discussed in the literature. Nielsen et al. (2017) investigated the effect of 3 weeks of BFR training (20%-1RM to concentric failure) compared to work-matched traditional resistance training performed at low (20%-1RM) or high-intensities (HI; 70%-1RM) and observed that proinflammatory macrophages (M1) increased 108% with BFR training and 165% with low intensity strength training 3 days after training cessation. Additionally, anti-inflammatory phenotype (M2) increased 163% only with BFR training. Membrane and intracellular HSP27 expression increased 60-132% at 8 days into the intervention with BFR. The authors concluded that signs of myocellular stress were observed in the early phase of the training intervention with inflammation occurring after the training program. Thus, this research furthers the hypothesis that the macrophage response to BFR training occurs due to a heightened inflammation response but is likely not related to muscle damage.

Regarding neutrophils, Behringer et al. (2017) investigated the effects of moderate-intensity (four sets at 75% of 1RM until volitional failure) eccentric knee extensions with BFR (20 mmHg) and without on blood lactate, endocrine response, muscle swelling, biomarkers of muscle damage, and neutrophil counts. The results showed that the total number of repetitions was lower in BFR compared with the control condition, however, despite a lower volume, BFR induced a similar increase in metabolic stress (lactate), muscle swelling, plasma GH, creatine kinase (CK), and neutrophil counts. The authors suggested that the lower oxygen supply during BFR may have led to a greater metabolic stress and membrane damage due reactive oxygen species (ROS) from an ischemia–reperfusion sequence or by invading neutrophils. The higher intensity (75% 1RM vs. 20-30% 1RM) and lower occlusion pressure may explain the discrepancies in markers of muscle damage found in this study compared to other BFR studies.

The mechanism by which macrophages and neutrophils are recruited to skeletal muscle after BFR is not fully understood, but can be partially attributed to transcription factors such as HIF-1α that respond to decreases in available oxygen at the cellular level (Drummond et al., 2008). Leire et al. (2013) investigated the role of AKB-4924, an activator of HIF-1α, on neutrophil recruitment in mice. The results showed that AKB-4924 increased the recruitment of neutrophils to the site of inflammation induced by lipopolysaccharide (LPS) injection. When a high dose (5 μg) of LPS was injected in HIF-1α *knockout* mice, neutrophil recruitment to the injury site was reduced compared with control mice. HIF-1α activation via hypoxia contributes to increased expression of MCP-1 (also known as CCL2) (Larkin et al., 2012), which is a chemokine with potent macrophage recruitment and activating functions (Lu et al., 1998; Chazaud et al., 2003). Roseguini et al. (2010) demonstrated that intermittent pneumatic compressions, a method conceptually similar to BFR, increases skeletal muscle MCP-1 expression in rats. It is likely the myocellular macrophage infiltration after BFR as demonstrated by Nielsen et al. (2017) may be explained by increased MCP-1 in skeletal muscle via HIF-1 activation. Thus, activation of HIF-1α may be one potential mechanism that BFR increases neutrophil and macrophage response.

BFR training increases the IL-6 production, which may also induce the recruitment of immune cells (Takarada et al., 2000; Patterson and Ferguson, 2011; Nielsen et al., 2017), and in particular, neutrophils. Fielding et al. (2008) showed that mice with alterations in IL-6/gp130 signaling by the knockout of IL-6 receptor (gp130) decreased the rate of neutrophil trafficking during acute inflammation. In addition, the researchers demonstrated that IL-6 can regulate neutrophil trafficking in a STAT3 dependent-manner. Therefore, increasing IL-6 levels via BFR training could increase neutrophil recruitment for skeletal muscle remodeling.

Another mechanism whereby BFR may increase the recruitment of neutrophils without inducing muscle damage may be attributed to catecholamine release, specifically norepinephrine. Takarada et al. (2000) demonstrated that a BFR protocol using five sets at 20% of 1-RM and 14 repetitions induced greater norepinephrine production compared with the same exercise protocol without occlusion. Shimizu et al. (2016) compared the influence of BFR (3 sets at 20% of 1RM with 30 s of rest interval) vs. resistance exercise without occlusion (control) on the endocrine response in healthy elderly people. It was observed that BFR generated a higher production of norepinephrine as well as lactate, VEGF and GH compared with the control condition. Previous studies showed that noradrenaline may act as “danger signals” during fatiguing exercise, thereby increasing neutrophil activation and function. Hinchado et al. (2012) found that after exercise when neutrophils were incubated with norepinephrine there was an increase of chemotaxis, phagocytosis and microbicide capacity, demonstrating an improved neutrophil function. These findings may explain in part the increase of neutrophils counts induced by BRF (Behringer et al., 2017), as norepinephrine seems to play a significant role in neutrophil function and activation.

The immune system appears to act in a straightforward manner to contribute to the plasticity of skeletal muscle tissue via the phagocytosis of foreign particles and increasing inflammation by releasing cytokines that stimulate further infiltration of immune cells in an attempt to repair and regenerate damaged tissues. We believe that hypoxia, metabolic mediators, and IL-6 mediated by BFR during low-intensity strength training could be important factors to potentiate the macrophage and neutrophil recruitment contributing to improvement skeletal muscle remodeling; however, there are few studies in the literature investigating the effects of BFR training on immune cells response according showed at Table 1. Therefore, future studies should be conducted to better understand the immune system response during acute and chronic BFR training, and to further investigate how BFR may be employed as an appropriate treatment in muscle wasting disorders characterized by chronic low-grade inflammation.

**Table 1:** Effect of blood flow restriction plus low-intensity strength training on the inflammatory response.

**Author (year)**	**Aim**	**Results**
Takarada et al., 2000	Compared the IL-6 response after BFR using five sets of 14 repetitions at 20% of 1-RM vs. the same exercise protocol without occlusion (control)	↑ IL-6, lactate, GH, and norepinephrine in BFR condition ↔ Creatine kinase in both conditions
Patterson and Ferguson, 2011	Compared the effects of BFR (five sets of unilateral knee extensions with 20% 1-RM and 30-s rest between each set) with low-load resistance exercise without BFR on the inflammatory response	↑ IL-6 in both conditions ↑ GH and VEGF only in BFR condition
Nielsen et al., 2017	Investigated the effect of 3 weeks of BFR training (20%-1RM to concentric failure) compared to two groups that performed a matched amount of work at low (20%-1RM) or high-intensities (HI; 70%-1RM)	↑ macrophages (M1) in BFR and low intensity training conditions ↑ macrophage (M2) only in BFR condition ↑ HSP27 expression only in BFR condition ↔ L-6, TNF-α, and MCP-1 in BFR condition ↑ CK only in HI condition
Behringer et al., 2017	Investigated the effects of moderate-intensity (four sets at 75% of 1RM until volitional failure) eccentric knee extensions with BFR (20 mmHg) and without (control) on blood lactate, hormonal response, muscle swelling, biomarkers of muscle damage and neutrophil counts	↓ volume in BFR compared with control condition ↑ neutrophil counts, lactate muscle swelling, GH, and CK in both conditions

*↑, significant increased; ↓, significant decreased; ↔, no changed*.

## Future perspective

The immune system is virtually involved in every repair and regeneration response in skeletal muscle cells. However, the immune system is not activated only in response to conditions of muscle damage, but also in response to muscle contractions, with muscle metabolites and substrates utilization being major drivers of such response. At the moment, several studies that demonstrate minimal to no muscle damage following BFR contractions refute the muscle damage hypothesis (Wilson et al., 2013; Loenneke et al., 2014b; Sudo et al., 2015). However, a certain degree of caution should be taken when interpreting this data since signs of muscle damage may occur in the absence of either soreness, reduced force production, and/or increased plasma CK. Nevertheless, infiltration of immune cells in the muscle is seen after a BFR exercisewhich poses the question of, mechanistically, who is directing immune cells to the muscle and what tasks they are performing, since there does not appear to be any measurable muscle damage. In regards to “who” is recruiting immune cells to the skeletal muscle, a multitude of mechanisms may exist, from cytokines secreted by the contracting muscle to activation of transcription factors activating chemotactic proteins (Giudice and Taylor, 2017; Vannella and Wynn, 2017), both of which are reinforced by muscle hypoxia. The question of “what task” they are performing may be more related to muscle remodeling, since BFR is a potent method to induce muscle hypertrophy, and, in fact, the studies which show a robust infiltration of immune cells to the muscle are the studies reporting robust muscle hypertrophy in short periods of time (i.e., 19 days) (Nielsen et al., 2012). Although classical hypertrophic pathways, such as mTOR, have been shown to be activated by BFR, non-canonical activations via growth factors secreted by the immune cells may reinforce the intrinsic muscle contraction effects on signaling pathways. Such cell signaling activation can lead to increased protein synthesis and muscle hypertrophy. In this case, multiple pathways and modus operandis may be coordinately working to produce muscle hypertrophy and immune system activation. The question remains: If there is no muscle damage, then what is triggering the immune system to work so hard in the BFR paradigm?

## Conclusion

Skeletal muscle hypertrophy is an important goal for individuals pursuing both athletic or rehabilitative gains. With this goal in mind, new strategies have been employed to investigate varying cell signaling pathways, cellular types, and hormonal triggers, and which seem to be activated via tension or metabolic overload. Traditionally, exercises involving muscle damage are well known activators of muscle satellite cells, pro-inflammatory cytokines, and immune cells. However, the recent literature has pointed out that metabolic stimuli (induced by resistance training plus BFR) such as hypoxia and metabolic overload (H^+^, P_i_, lactate accumulation) are also potential activators of IL-6, macrophages and neutrophils. Taken collectively, these anabolic stimuli could help to explain the effectiveness of such methods in the absence of high mechanical forces and muscle damage. Mechanistic studies will help to dissect the importance of each variable in the role of the metabolic response, thus strengthening the concept that BFR exercises, although characterized as low force exercises, are effective not only to increase the MPS response, but also the multitude of immune cell responses that accompanies muscle hypertrophy.

## Author contributions

FR, MdF, NZ, FL, and JC conceptualized and drafted the paper. All authors revised the work and final approval of the manuscript.

### Conflict of interest statement

The authors declare that the research was conducted in the absence of any commercial or financial relationships that could be construed as a potential conflict of interest.
